# An Unusual Presentation of Ludwig's Angina Complicated by Cervical Necrotizing Fasciitis: A Case Report and Review of the Literature

**DOI:** 10.1155/2012/931350

**Published:** 2012-08-02

**Authors:** Kristelle Chueng, David J. Clinkard, Danny Enepekides, Yousef Peerbaye, Vincent Y. W. Lin

**Affiliations:** ^1^Department of Otolaryngology-Head & Neck Surgery, Sunnybrook Health Sciences Centre, Toronto, ON, Canada M4N 3M5; ^2^Department of Emergency Medicine, Sunnybrook Health Sciences Centre, Toronto, ON, Canada M4N 3M5

## Abstract

Ludwig's angina can seldom be complicated by necrotizing fasciitis. Due to the rapidly progressing nature of this infection and the potential for airway compromise and death, it is important to be aware of different ways in which this disease process can present in order to recognize and treat it emergently. We report here an unusual presentation of a case of Ludwig's angina complicated by necrotizing fasciitis in an elderly patient. The clinical features, diagnosis, and treatment are discussed in detail as well as a brief literature review on craniocervical necrotizing fasciitis.

## 1. Introduction

Ludwig's angina is a rapidly progressing necrotizing cellulitis affecting the posterior oropharynx, submaxillary, and sublingual spaces [1]. It usually arises following a dental extraction or infection and is potentially fatal due to airway obstruction [2]. Patients may present with dysphagia, trismus, drooling, and shortness of breath [2]. The management of Ludwig's angina involves antibiotics, maintenance of a secure airway to prevent asphyxia, and surgical drainage if necessary [3]. Infrequently, Ludwig's angina has been documented to extend deeper into the soft tissues and progress to craniocervical necrotizing fasciitis (CCNF). 

Necrotizing fasciitis is a rare soft tissue infection resulting in the death of subcutaneous and fascial tissue [4]. The infection spreads along the fascial planes and can extend into surrounding vessels, nerves, and muscle tissue [5]. It usually occurs in the extremities, abdomen, perineum, and rarely in the head and neck [4]. In most cases of craniocervical necrotizing fasciitis, the original source of the infection is odontogenic, usually an undiagnosed or treated dental abscess [6]. Alternatively, CCNF has been documented to result from periodontic disease, tooth impaction or failed tooth extraction [7, 8]. CCNF is thought to be caused by polymicrobial infection, with both facultative aerobic and anaerobic bacteria being implicated (e.g., Peptostreptococcus, Staphylococcal species, and Streptococcal species) [7]. Predisposing factors that have been identified in association with CCNF include extremes of age, immunosuppression, diabetes mellitus, alcohol, and tobacco smoking [4, 7]. 

Histopathology confirms the diagnosis of necrotizing fasciitis but early recognition of CCNF can usually be achieved based on a combination of clinical evaluation and laboratory investigations [4, 7]. The clinical disease process usually begins with painful edema and erythema followed by a purplish discolouration of the skin, gas-filled bullae, pus formation, and eventually frank necrosis of the affected area. In the early stages of the disease, it may be difficult to differentiate between CCNF and nonnecrotizing soft tissue infections such as cellulitis or erysipelas. Although there are no clinical features that are pathognomonic for CCNF, certain physical signs increase its likelihood. Pain, hyperpyrexia, and tachycardia that are out of keeping with the degree of soft tissue involvement are more suggestive of CCNF. Furthermore, anesthesia of the affected area as a result of nerve involvement is an early sign of NF. Certain laboratory values (WBC > 14 × 109/L, serum Na < 135 mmol/L, blood urea > 15 mg/dL) have also been found to be associated with CCNF, although they are nonspecific for this disease entity. 

These clinical and laboratory findings are useful in formulating a presumptive diagnosis of necrotizing fasciitis, but they are not always present. The following case is an example of an atypical presentation of necrotizing fasciitis that progressed from Ludwig's angina.

## 2. Case Report

An 80 year-old healthy female presented to the emergency department following a two-day history of dysphagia. On initial examination, the patient was leaning forward with her head in a sniffing position, her tongue was protruding, and soft but audible stridor could be heard. She was afebrile and had a heart rate of 84, blood pressure of 170/70, respiratory rate of 18, and an oxygen saturation of 96% on room air. Examination revealed anterior neck fullness with a deep purplish discoloration of her skin. There was significant swelling, and induration in the submandibular and submental regions extending down towards the base of neck. No major cartilaginous landmarks could be palpated. Inferiorly, the sternal notch could be palpated. Intraoral exam demonstrated some mild trismus, swelling, and purplish discolouration of the base of the tongue, and a very firm and woody floor of mouth bilaterally. Bloodwork showed WBC (12.2), Na (141) and BUN (22.2), creatinine (226), and CK (787, reference is <170) values. She was administered intravenous antibiotics and supplemental oxygen. 

**Figure 1:**
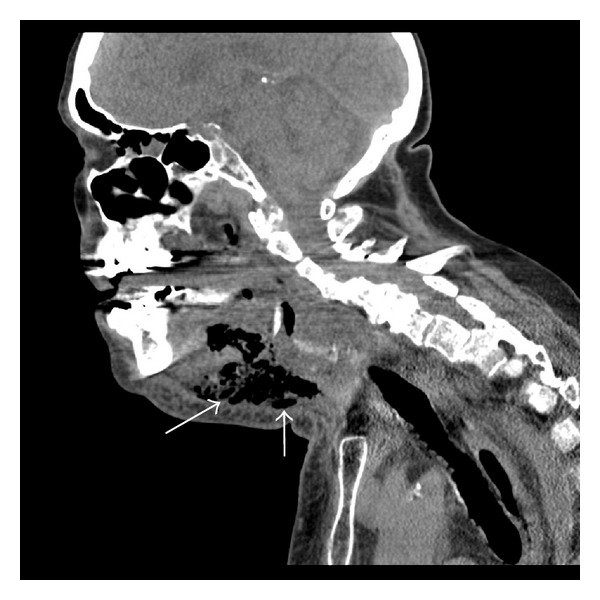
Sagittal CT image of patient preoperatively. Note the classic “sniffing” position of patient to maximize air entry in the presence of an upper airway obstruction. White arrows point to significant gas formation in anterior cervical space.

**Figure 2:**
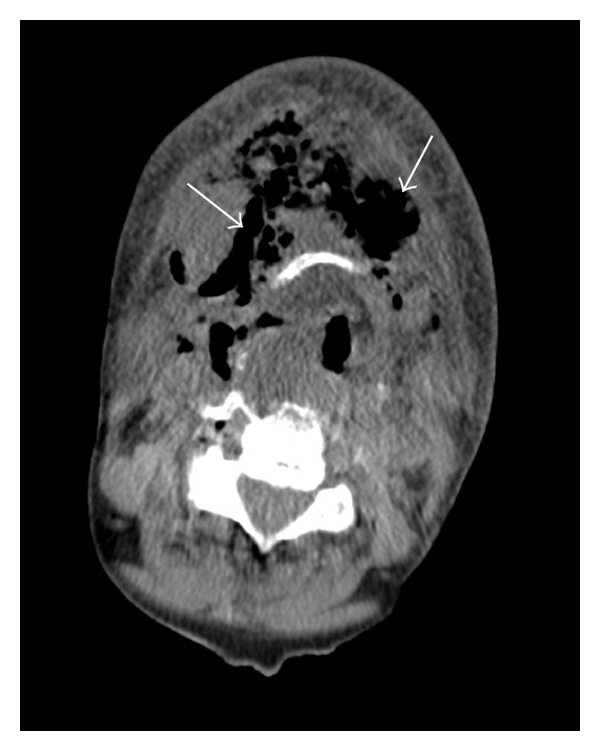
Axial CT image of patient preoperatively at level of hyoid. Note again presence of gas in anterior cervical space and stranding of fat suggestive of acute infection.

A flexible nasopharyngoscopy was performed and significant airway edema including the tongue base and epiglottis was noted. A CT scan was obtained which revealed significant gas in anterior cervical space suggestive of infection (Figures 1 and 2).

 Following transfer to the OR, surgical airway and debridement was attempted. When sitting upright, the patients anterior neck was infiltrated with xylazine and an awake tracheostomy was commenced. Immediately after the incision was made, a large amount of purulent and necrotic tissue was encountered. Blunt tissue dissection was carried out until the anterior wall of the trachea was palpated. At that point the patient began to desaturate. An urgent transverse linear incision was carried out to enter the trachea and an endotracheal tube inserted and inflated. End-tidal CO2 was immediately obtained and the patient was manually ventilated until her saturations which were as low as 30% for a brief period of time stabilized at 99%. She was then placed under general anaesthesia and an extensive debridement of her anterior neck, bilateral submandibular and sublingual spaces were carried out. 

**Figure 3:**
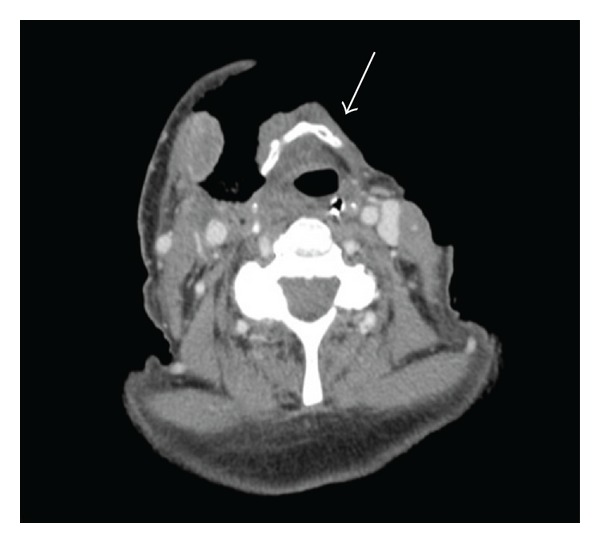
Postoperative CT scan at level of the hyoid. Note: the extensive loss of soft tissue in the anterior neck compartment (arrow).

Intraoperative pathology of the necrotic tissue confirmed the diagnosis of Ludwig's angina which then developed into necrotizing fasciitis of her neck. Postoperative CT scans demonstrate clear loss of anterior neck soft tissue (Figure 3). 

**Figure 4:**
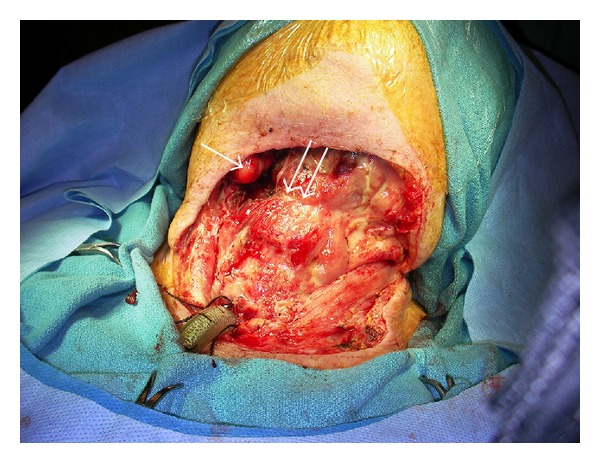
Intraoperative image taken after repeat debridement. Note: exposed submandibular gland on right side (single arrow) and exposed hyoid bone (double arrow).

While recovering in the ICU, the patient developed acute renal failure and hypotension, which ultimately stabilized. Further debridements were necessary to completely remove the necrotic tissue (Figure 4) and the impacted mandibular molar responsible for the infection was subsequently extracted by oral surgery. Three weeks after admittance, a pedicled deltopectoral flap was used to reconstruct her soft tissue defect. The tracheostomy was eventually decannulated and the patient was discharged home.

## 3. Discussion

This paper illustrates an interesting scenario in which a patient with prior dental surgery developed an atypical presentation of Ludwig's angina complicated by necrotizing fasciitis. Although she did present with signs of Ludwig's angina (i.e., dysphagia and respiratory distress), the only sign of concomitant necrotizing fasciitis was the edema and purplish discolouration of the skin overlying her neck region. She did not present with many of the other characteristic clinical findings (e.g., tachycardia, fever, and severe pain) or laboratory findings (e.g., very elevated WBC and low serum sodium) associated with typical necrotizing fasciitis. This demonstrates the often different presentation of illness in the elderly and the importance of maintaining a high index of suspicion for necrotizing fasciitis in the setting of Ludwig's angina. 

Imaging modalities can assist in the diagnosis of necrotizing fasciitis if it is not clinically apparent. Certain findings on a CT scan increase the likelihood of necrotizing fasciitis such as the inflammation of skin and subcutaneous fat, the involvement of more fascia than muscle, and the presence of gas gangrene in the superficial fascia [7]. Gas formation was evident on the preoperative head and neck CT scans (Figures 1 and 2) in the anterior cervical region of the patient in the case, which helped to support the diagnosis of CCNF. On an MRI scan, necrotizing fasciitis appears hyperintense in the fascial planes on T2-weighted images, and appears as an area of hypointense attenuation that does not enhance with contrast on T1-weighted images [7]. 

The mainstay of treatment for CCNF involves surgical debridement of necrotic tissue and empiric broad-spectrum intravenous antibiotics followed by culture-based antibiotics [7, 9], as was done for the patient in the case. It is worth noting that if she had not been in a centre where operative intervention was possible, securing the airway prior to transport would have presented significant challenges. The optimum way of managing her airway in such a scenario would have been via awake bronchoscopic intubation. 

Hyperbaric oxygen therapy can be administered as adjunctive therapy although its use is controversial and further studies are required to support its effectiveness in treating necrotizing fasciitis [10]. Thoracotomy and drainage can be performed in cases of CCNF complicated with a mediastinal abscess [11]. Treatment should begin as soon as a diagnosis is made to prevent further spread of infection. Early and aggressive management is associated with decreased mortality [7]. 

The complications of CCNF are both local and systemic. Direct and distant spread of the infection can lead to complications that include intracranial, retropharyngeal, and pulmonary infections. Hematogenous dissemination can also occur, leading to complications such as septic shock, rheumatic disease, and cardiac problems [12]. If left untreated, the rapid dissemination of the infection can be fatal. Necrotizing fasciitis carries an average mortality rate of about 30% [13].

## 4. Conclusions

Ludwig's angina complicated by craniocervical necrotizing fasciitis can present without the expected clinical or laboratory findings. Since delayed diagnosis is associated with high mortality, doctors must maintain a high index of suspicion for necrotizing fasciitis when managing a patient with Ludwig's angina. As soon as imaging modalities corroborate the CCNF findings on clinical exam, the initial goals of management should be to secure the airway and perform aggressive surgical debridement. Craniocervical necrotizing fasciitis is a surgical emergency that can be successfully treated with appropriate and early intervention.
